# Correction to European Academy of Neurology (EAN)/European Federation of Autonomic Societies (EFAS)/International Neuro‐Urology Society (INUS) Guidelines for Practising Neurologists on the Assessment and Treatment of Neurogenic Urinary and Sexual Symptoms (NEUROGED Guidelines)

**DOI:** 10.1111/ene.70205

**Published:** 2025-05-13

Panicker, J.N., Fanciulli, A., Skoric, M.K., Kaplan, T., Aleksovska, K., Adamec, I., Averbeck, M.A., Campese, N., Guaraldi, P., Leys, F., Moreno‐Palacios, J., Simeoni, S., Stankovic, I., Wright, S., Batla, A., Blok, B., Hentzen, C., Hilz, M.J., Kessler, T.M., Madersbacher, H., Nair, K.R., Nair, K.P.S., Pakzad, M., Traon, A.P.‐L., Peryer, G., Przydacz, M., Sakakibara, R., Saraf, U., Smith, M., Struhal, W., Thijs, R.D., Tudor, K.I., Tutaj, M., Vodušek, D.B., Wenning, G., and Habek, M. (2025), European Academy of Neurology (EAN)/European Federation of Autonomic Societies (EFAS)/International Neuro‐Urology Society (INUS) Guidelines for Practising Neurologists on the Assessment and Treatment of Neurogenic Urinary and Sexual Symptoms (NEUROGED Guidelines). *Eur J Neurol*, 32: e70119. https://doi.org/10.1111/ene.70119.

In the above article, the following open access funding statement has been added: Open Access funding provided by Medizinische Universitat Innsbruck/KEMÖ.

We apologize for this error.

